# Gender, marginalised groups, and young people’s mental health: a longitudinal analysis of trajectories

**DOI:** 10.1186/s13034-024-00720-4

**Published:** 2024-02-28

**Authors:** Suzet Tanya Lereya, Sam Norton, Maddison Crease, Jessica Deighton, Angelika Labno, Giulia Gaia Ravaccia, Kamaldeep Bhui, Helen Brooks, Cara English, Peter Fonagy, Margaret Heslin, Julian Edbrooke-Childs

**Affiliations:** 1 Evidence-Based Practice Unit, University College London and Anna Freud, London, UK; 2 Department of Psychology and Centre for Rheumatic Diseases, King’s College London, London, UK; 3 Anna Freud, London, UK; 4 Department of Psychiatry & Nuffield Department of Primary Care Health Sciences Senior Research Fellow, Wadham College, University of Oxford, Oxford, UK; 5 Division of Nursing, Midwifery and Social Work, School of Health Sciences, Faculty of Biology, Medicine and Health, University of Manchester, Manchester Academic Health Science Centre, Manchester, UK; 6 College of Arts, University of Glasgow and Gendered Intelligence, London, UK; 7 Anna Freud Centre & Department of Clinical Educational and Health Psychology, University College London, London, UK; 8 Health Service & Population Research, Institute of Psychiatry, Psychology & Neuroscience, King’s College London, London, UK

**Keywords:** Adolescent, Female, Mental Health, Minority groups

## Abstract

**Background:**

Individuals from marginalised groups experience higher levels of mental health difficulties and lower levels of wellbeing which may be due to the exposure to stress and adversity. This study explores trajectories of mental health over time for young women and girls and young people with other marginalised identities.

**Methods:**

We conducted a secondary analysis on *N =* 14,215 children and young people (7,501 or 52.8% female, 6,571 or 46.2% male, and 81 or 0.6% non-binary or questioning) who completed a survey at age 11 to 12 years and at least one other annual survey aged 12 to 13 years and/or aged 13 to 14 years. We used group-based trajectory models to examine mental health difficulties.

**Results:**

Except for behavioural difficulties, young women’s and girls’ trajectories showed that they consistently had higher levels of mental health difficulties compared to young men and boys. A similar pattern was shown for non-binary and questioning children and young people. Children and young people with economic disadvantage and/or special education needs, and/or for whom there were welfare concerns, were generally more likely to experience higher levels of mental health difficulties.

**Conclusions:**

This information could inform public policy, guidance and interventions.

**Supplementary Information:**

The online version contains supplementary material available at 10.1186/s13034-024-00720-4.

There is a consistent pattern in the literature around the increasing levels of mental health difficulties for young women and girls. A cross-sectional survey of 15-year olds across 73 countries showed that adolescent young women reported lower levels of mental wellbeing than adolescent young men [1]. Another study showed that young women and girls, aged 11 to 12 years, have higher levels of mental health difficulties compared to young men and boys but these levels of difficulties increase year on year [2]. One in five children and young people in England, aged 8 to 25 years, experience mental health difficulties, a rise from one in ten in 2017 [3]. Moreover, in young people aged 17 to 19 years, the prevalence of a probable mental disorder was twice as high in young women (31.6%) as in young men (15.4%) [3]. The types of difficulties experienced differ for young women and girls versus young men and boys, with the former experiencing higher levels of internalising difficulties such as anxiety, phobia and depression and the latter experiencing higher levels of externalising difficulties such as oppositional defiant disorder and conduct disorder [4]. It has been suggested that gender differences in emotion expression are the result of a combination of biologically based temperamental predispositions and the socialisation of boys and girls [5]. In most cultures, girls are expected to display greater levels of happiness and to internalise negative emotions, such as sadness, fear, anxiety, shame, and guilt compared to boys [6, 7]. On the contrary, boys are generally expected to show less of sensitive emotions, and they are “allowed” to express externalising emotions such as anger [6]. These social expectations are in line with women’s traditional role as caregivers and males’ traditional roles as protector of their families [8]. However, when a person is limited in the range of emotions expressed or is encouraged to express particular emotions to the exclusion of others, there is a greater likelihood of compromised socioemotional functioning and heightened risk for developing certain mental difficulties [9].

These mental health differences may also be related to gender equality and cultural attitudes of gender equality. In a study of adult mental health, the gap has been found to be smaller in countries with greater gender equality [10]. Other evidence has however shown that, even in countries with more political, educational, economic, and health gender equality, gender differences in mental health persist or are even more pronounced [1]. In countries with greater gender equality, girls and women confront a “double burden” of navigating heightened economic and political engagement alongside the enduring expectations of traditional female responsibilities and norms. Despite women making strides into traditionally male-dominated employment sectors in more gender-equal nations, a comparable level of entry by men into female-dominated sectors is lacking. Additionally, the distribution of domestic responsibilities remains unequal, with men not contributing equivalent amounts of domestic work [1, 11, 12]. There is some evidence to indicate that gender inequalities may explain the relationships between social media and mental health for young people. One study found that chatting and self-presentation social media activities were associated with higher levels of internalising difficulties for girls but not boys [13].

Throughout the life course, women are more likely to face disadvantages resulting from societal structures that assign specific roles, which can drive disparities. These roles may include socio-economic deprivation (such as average earnings and workplace discrimination), childbearing and caregiving responsibilities, as well as experiences of discrimination, harassment, trauma, and abuse [14, 15]. The evidence is so compelling that the World Health Organization identifies gender as being a structural determinant of mental health [16]. Indeed, as the pandemic has increased not only housework, but also family responsibilities, including childcare needs primarily managed by women in response to school closures, young women, girls, and marginalised groups have been disproportionately affected by the COVID-19 pandemic [17–19].

Ethnicity is another important factor that impacts mental health difficulties. While not all minoritised ethnic groups exhibit higher levels of mental health difficulties, and findings can vary substantially across countries [20], research from the United Kingdom (UK) suggests that young people and adults from black and minoritised ethnic groups are more likely to experience certain severe mental illnesses and are more likely to be involuntarily admitted to hospital compared with those of white ethnicity [21, 22]. Other marginalised groups of children and young people who experience higher levels of mental health difficulties include those with higher levels of economic disadvantage, additional learning needs, child protection plans or child in need status (where authorities have identified concerns about a child’s safety) [23–25]. Members of lesbian, gay, bisexual, transgender, questioning, intersex, asexual, and other (LGBTQIA+) communities experience disproportionately high levels of mental health difficulties compared to cis-gendered, heterosexual individuals [26, 27].

According to minority stress theory, individuals from marginalised groups experience higher levels of mental health difficulties due to the exposure to stress and adversity arising from oppression, structural inequity, and systemic discrimination [28, 29]. From an intersectional perspective, individuals from multiple marginalised groups experience this mental health disadvantage for each marginalised identity, and the effect of the interplay between identities is greater than the combined effect of individual identities [30]. For example, an international study found that in countries with larger disparities between levels of pay in the population, immigrant young women and girls with higher levels of economic disadvantage had significantly lower levels of life satisfaction than expected from the addition of the individual effects of migration, gender identity, and economic disadvantage [31]. Additionally, the network theory of mental health difficulties suggest that symptoms of psychopathology are causally connected through biological, psychological, and societal mechanisms [32]. Statistical models have been computed to investigate whether activation of specific symptoms can spread throughout the network—a phenomenon referred to as the “connectivity hypothesis.” Indeed, it has been found that an “external stressor” can trigger a cascade of activation which persists even after the initiating stressor is removed [33]. Therefore, individuals from marginalised groups may persist in experiencing mental health challenges even after the removal of the initial stressor.

To address the rising levels of mental health difficulties for young women and girls, public mental health approaches are needed that tackle inequalities. These may promote social and political changes that disrupt the persistence of inequalities and offer new practices through which these inequalities can be eradicated at the macro level [15]. For example, addressing the demands placed on women through the traditional heteronormative organisation of the labour market and home-life domains, which negatively impact women’s mental health, whether due to the pressures of adherence or opposition. To inform these approaches, population research is needed to identify patterns of mental health difficulties for young women and girls over time.

The aim of the present study was to answer the overarching research question: What are the mental health trajectories for young women and girls? To do so, we examined four specific research questions:

1. Are there distinct groups of trajectories for mental health over three years?
2. Are young women and girls disproportionately represented in trajectory groups with higher levels of and/or steeper increases in mental health difficulties?
3. Which marginalised groups (gender, ethnicity, deprivation, special educational needs, care experience) are more likely to be represented in trajectory groups with higher levels of mental health difficulties?
4. Is there evidence for intersectional effects between gender and membership of other marginalised groups?

## Methods

### Data source

Data were collected for an evaluation of Headstart, a programme that focused on improving mental health difficulties and wellbeing, and preventing serious mental health difficulties, for children and young people aged 10 to 16 years (funded by the National Lottery Community Fund). For the present analysis, the dataset was collected between 2017 and 2019 from 104 schools from six local areas in England. The sample of schools was not drawn to be representative of all school children in England; it was based on local areas that were part of the HeadStart programme, and each of the local areas selected the schools to participate [34]. Every year, children and young people from specific year groups/grades in participating schools completed surveys using a secure online system during a usual school day as part of a teacher-facilitated session. Consent was obtained from parents/carers; children and young people provided assent prior to starting the survey, and ethical approval was received by the UCL ethics committee (reference: 8097/003).

### Participants

Out of the 15,476 children and young people who completed the survey in year 7 (ages 11 to 12), the analyses reported are based on *N =* 14,215 children and young people who completed at least one other annual survey in year 8 (ages 12 to 13) and/or year 9 (ages 13 to 14). Compared to the national average, the included sample had a slightly higher proportion of children and young people with higher levels of economic disadvantage, based on eligibility to receive free school meals (study sample: 16.3%, national average: 12.9%). The study sample had a lower proportion of children and young people with support for special educational needs (study sample: 11.8%, national average: 14.4%) and a slightly higher proportion of white children and young people (study sample: 76.0%, national average: 75.2%).

### Measures

#### Socio-demographic characteristics

The socio-demographic characteristics of children and young people were extracted through data linkage with the National Pupil Database (NPD), which comprises socio-demographic and education data on all children and young people in all schools in England.

Gender identity was extracted from the NPD. As this captured female vs. male, we supplemented the NPD data with self-reported gender identity for *n =* 2,742 young people who completed the year 11 survey, which had asked about gender identity and had an inclusive range of response options. These two sources resulted in three gender identities: female (cis and trans young women and girls), male (cis and trans young men and boys), and non-binary or questioning. We recognise non-binary and questioning are not the same gender identities; however, we chose to combine them so that we were able to represent diverse gender identities in the analysis. We also recognise that those for whom data were extracted from the NPD may have chosen another identity than female vs. male if they had been asked. We chose to retain the survey responses for the small sample so that we could prioritise the voice of participants who had self-identified and to represent diverse genders, even if not for the entire sample.

From the outset of the study, we worked with young people advisors, who highlighted the importance of using an inclusive definition of gender identity. Hence, while it was possible to distinguish between cis and trans young people (i.e., they were asked whether their gender was different from the sex they were assigned at birth), we have chosen to prioritise young people’s description of their gender. Young people could also choose to not respond to the self-reported gender identity question. We retained these responses in the analysis but do not report them because including this as a category of gender identity, when a person chose to not identify a gender, was conceptually inconsistent. We define gender identity as an aspect of who an individual is, how they are seen, and how they interact with the world around them.

Ethnicity, free school meal eligibility, special educational needs, and child in need status at baseline were extracted from the NPD. Ethnicity was grouped into five broad groupings (Asian, black, mixed race, “other” ethnic groups, and white). Whether a child or young person had ever being eligible to receive free school meals (0 = no, 1 = yes) is frequently used as an indicator of economic disadvantage as only families on income support are eligible. The presence of special educational needs (0 = no, 1 = yes) refers to formally identified needs. The presence of a child in need status (0 = no, 1 = yes) refers to social care needs, most frequently because of concerns about abuse or neglect, acute family stress, or family functioning.

#### Mental health difficulties

To measure mental health difficulties, the 25-item self-reported Strengths and Difficulties Questionnaire [SDQ; 35] was used. It comprises four difficulties scales (emotional difficulties, behavioural difficulties, hyperactivity and/or inattention difficulties, and difficulties with peer relationship) and a strengths-based prosocial behaviour scale. Each sub-scale has 5 items and each item of the SDQ is scored on a 3-point scale with 0 = not true, 1 = somewhat true and 2 = certainly true. Emotional difficulties and behavioural difficulty scores are created by combining 5 items of the respective subscales and scores ranged from 0 to 10, with higher scores indicating higher levels of difficulties. A total of the four difficulties subscales (20 items) are used to create an overall total difficulties score, with scores ranging from 0 to 40 with higher scores indicating higher levels of difficulties. We focussed on the emotional and behavioural difficulties scores, as they were of particular relevance to the research questions, and the total difficulties score, which also includes the remaining two subscales. The SDQ is a widely used measure with evidence of reliability and validity [36]. For the current study, the Cronbach’s alphas for total difficulties were 0.81, 0.82, and 0.82 for the first, second, and third years, respectively.

### Analytic strategy

#### RQ 1: are there distinct groups of trajectories for mental health over time?

Group-based trajectory models were used to determine whether distinct trajectory groups of mental health difficulties could be recovered from three time points between year 7 (ages 11 to 12) and year 9 (ages 13 to 14). Group-based trajectory models allow clusters of individuals who share similar trajectories over time to be identified [37]. The group-based trajectories over time were modelled in Stata 17 (StataCorp, 2021, College Station, Texas, USA) using the plugin Stata Traj [38]. Detailed documentation of the Traj procedure can be found at https://www.andrew.cmu.edu/user/bjones/.

We deviated from the pre-registered protocol by analysing data spanning three years (2017/18, 2018/19, 2019/20) instead of the planned four years (including 2020/21). This adjustment was made due to data availability constraints. Additionally, our focus narrowed to mental health difficulties exclusively, omitting wellbeing from the scope for increased precision.

Models were estimated separately for total difficulties, and then emotional and behaviour difficulties for a more detailed examination. To determine the number of trajectory groups that best fit the data, we fitted between one and nine trajectories for each outcome, using quadratic polynomial terms to allow for non-linear trajectories to be recovered. In the instance where there were estimation problems due to the polynomial terms, these terms were removed from the model for the groups where the terms were indicated to be problematic. As a sensitivity check, the solution was verified against the model with the same number of classes, but only linear terms were specified to test interpretation. We used the Bayesian Information Criterion (BIC) as a fit index for selecting the best fitting model [39, 40], where BIC values closest to zero denote a better fitting model. However, because BIC sometimes keeps improving (decreasing) when adding trajectory groups [37], we considered a model inferior when a trajectory group contained less than 5% of the sample and when the model no longer captured new distinctive features of the data [41]. The degree to which the models were able to classify children and young people into different groups was assessed using entropy and average posterior probabilities of class membership [37].

#### RQ 2: are young women and girls more likely to be represented in trajectory groups with higher levels of, and/or steeper increases in, mental health difficulties?

Once the solution with the optimal number of groups was selected, the mental health difficulties models were extended to incorporate the estimation of socio-demographic characteristics that may be associated with the probability of a group trajectory. For gender identity, male was selected as the reference category as the focus of the analysis was young women and girls. For ethnicity, white was selected as the reference category as it was the largest group. The use of full-information maximum likelihood meant that all children and young people providing outcome data for the first wave of data collection and at least one of the other assessments were retained in the analysis, under the assumption that data were missing at random (i.e., conditional on variables associated with missingness being included in the model).

The analysis was conducted using Stata 17.

#### RQ 3: which marginalised identities are more likely to be represented in trajectory groups with higher levels of mental health difficulties?

The trajectory models for the mental health difficulties included ethnicity, free school meal eligibility, special education needs status, and child in need status.

#### RQ 4: is there evidence for intersectional effects between gender and membership of other marginalised groups?

Multinomial logistic regressions were conducted predicting group membership (using the identified solution) with the socio-demographic characteristics and interaction terms between gender (female, male) and the remaining socio-demographic characteristics (ethnicity, free school meals, special educational needs, child in need). Due to the small number of individuals identifying as non-binary or questioning, it was not possible to examine interaction effects with predictors for this group. Models were weighted by the probability of class membership to account for classification uncertainty.

## Results

Sample characteristics are shown in Table 1. We present the findings of the mental health difficulties trajectories by research question.

**Table 1:** Sample characteristics

	Male (*n*, %)	Female (*n*, %)	Non-binary or questioning (*n*, %)	Total (*n*, %)
**Gender** ^**a**^	6,571 (46.2%)	7,501 (52.8%)	81 (0.6%)	
**Ethnicity**				
Asian	536 (8.5%)	829 (11.2%)	< 10	N/A
Black	376 (6.0%)	418 (5.7%)	< 10	N/A
Mixed	254 (4.0%)	295 (4.0%)	< 10	N/A
Other	212 (3.4%)	215 (2.9%)	< 10	N/A
White	4,923 (78.1%)	5,609 (76.2%)	58 (78.4%)	10,637(77.1%)
**Ever being eligible to free school meals until 2016/17**				
No	4,065 (64.5%)	4,782 (64.9%)	50 (67.6%)	8,942 (64.8%)
Yes	2,236 (35.5%)	2,584 (35.1%)	24 (32.4%)	4,858 (35.2%)
**Special education need status**				
No	5,199 (83.8%)	6,723 (91.9%)	62 (84.9%)	12,039 (88.2%)
Yes	1,003 (16.2%)	591 (8.1%)	11 (15.1%)	1,609 (11.8%)
**Child in need status**				
No	5,958 (94.6%)	6,999 (95.0%)	68 (91.9%)	N/A
Yes	343 (5.4)	367 (5.0)	< 10	N/A
**Total difficulties at baseline – Mean (SD), n**	13.55 (6.34), 6,525	12.88 (6.39), 7,452	14.51 (6.19), 81	13.21 (6.38), 14,120
**Emotional difficulties at baseline – Mean (SD), n**	3.32 (2.39), 6,532	4.25 (2.52), 7,461	4.31 (2.48), 81	3.82 (2.50), 14,136
**Behavioural difficulties at baseline – Mean (SD), n**	2.82 (2.10), 6,536	2.12 (1.92), 7,467	2.43 (1.86), 81	2.45 (2.03), 14,146

*Note. N =* 14,215. ^a^ = participants who chose to not report gender are not shown. Frequencies < 10 have been suppressed and totals that include < 10 are not included so that the frequencies not calculated for the < 10 cases

### RQ 1: are there distinct groups of trajectories for mental health over time?

**Fig. 1:**
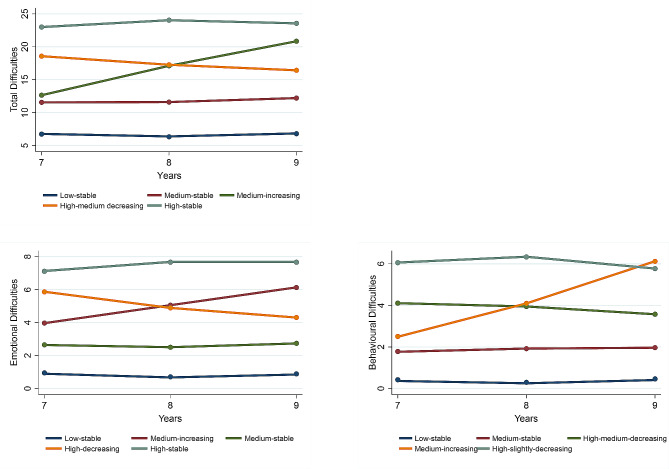
Trajectories of mental health difficulties for the 5-class model *Note: Year 7 (aged 11/12); Year 8 (aged 12/13); Year 9 (aged 13/14)*. Total difficulties: (1) low-stable (3,113/14,215; 21.9%), (2) medium-stable (5,305/14,215; 37.3%); (3) medium-increasing (1,366/14,215; 9.6%), (4) high-medium decreasing (3,105/14,215; 21.8%), (5) high-stable (1,325/14,215; 9.3%). Emotional difficulties: (1) low-stable (1,470/14,215; 10.3%), (2) medium-increasing (3,010/14,215; 21.2%), (3) medium-stable (5,762/14,215; 40.5%), (4) high-decreasing difficulties (2,118/14,215; 14.9%), (5) high-stable difficulties (1,855/14,215; 13.0%). Behavioural difficulties: (1) low-stable (2,066/14,215; 14.5%), (2) medium-stable (7,220/14,215; 50.8%), (3) high-medium-decreasing (3,538/14,215; 24.9%), (4) medium-increasing difficulties (532/14,215; 3.7%), (5) high-slightly-decreasing (860/14,215; 6.0%)

Based on the BIC, retaining models where each group contained at least 5% of observations, and models providing useful explanatory power, the 5-class solution was selected as being optimal, which are show Table 2. It is important to note, however, that models with 3-, 4-, or 5-classes could be considered optimal by different metrics (see Table S1, available online). As the 3- and 4-class models included only stable trajectories and did not recover subgroups with either increasing or decreasing trajectories, we focused on 5-class models (see Fig. 1 for 5-class model). Sensitivity analysis fitting a 5-class total difficulties model separately to the sample of females and males showed that the model from the total sample was well replicated, with only small differences in estimates of the intercepts and slopes for most trajectories (see Figure S2, available online). The average posterior class membership probabilities for the 5-class model ranged from 0.65 to 0.83, indicating good fit.

**Table 2:** Odds ratios from the 5-class traj models

	Total difficulties			Emotional difficulties			Behavioural difficulties		
	OR	95% CI	*p-*value	OR	95% CI	*p-*value	OR	95% CI	*p-*value
**Group**	**2: Medium trajectory group**			**2: Medium-increasing trajectory group**			**Group 2: Medium trajectory group**		
Female ^a^	1.08	0.94–1.24	0.282	13.68	10.51–17.81	0.000	0.63	0.55–0.73	0.000
Non-binary or Questioning ^a^	2.58	0.63–10.48	0.187	27.32	4.68–159.39	0.000	0.42	0.17–1.01	0.053
Ethnicity - Asian ^b^	0.77	0.63–0.93	0.006	0.50	0.35–0.71	0.000	1.19	0.96–1.47	0.105
Ethnicity - Black ^b^	0.82	0.63–1.06	0.132	0.22	0.14–0.34	0.000	1.87	1.29–2.72	0.001
Ethnicity - Mixed ^b^	0.78	0.56–1.08	0.130	0.58	0.36–0.92	0.021	1.02	0.71–1.45	0.929
Ethnicity - Other ^b^	0.81	0.57–1.16	0.260	0.52	0.28–0.95	0.033	1.56	0.98–2.51	0.063
Free school meal eligibility (yes) ^c^	1.42	1.22–1.66	0.000	1.53	1.23–1.91	0.000	1.87	1.58–2.21	0.000
Special education need (yes) ^d^	1.89	1.42–2.51	0.000	1.49	1.02–2.17	0.037	1.87	1.36–2.56	0.000
Child in need status (yes) ^e^	1.10	0.76–1.59	0.605	0.74	0.47–1.18	0.210	1.15	0.75–1.76	0.513
**Group**	**3: Medium-increasing trajectory group**			**3: Medium trajectory group**			**3: Medium/high-decreasing trajectory group**		
Female ^a^	2.45	2.01–3.00	0.000	2.56	2.07–3.18	0.000	0.31	0.26–0.37	0.000
Non-binary or Questioning ^a^	14.67	4.11–52.32	0.000	1.27	0.13–12.40	0.838	0.40	0.15–1.05	0.061
Ethnicity - Asian ^b^	0.32	0.23–0.45	0.000	1.01	0.76–1.33	0.969	0.64	0.48–0.86	0.003
Ethnicity - Black ^b^	0.30	0.18–0.49	0.000	0.49	0.36–0.66	0.000	1.72	1.16–2.55	0.007
Ethnicity - Mixed ^b^	0.69	0.46–1.03	0.072	0.79	0.53–1.18	0.251	0.86	0.57–1.30	0.465
Ethnicity - Other ^b^	0.58	0.36–0.94	0.028	0.95	0.58–1.55	0.822	1.31	0.80–2.14	0.287
Free school meal eligibility (yes) ^c^	2.25	1.86–2.72	0.000	1.28	1.05–1.56	0.014	3.43	2.86–4.12	0.000
Special education need (yes) ^d^	2.29	1.64–3.22	0.000	1.38	1.01–1.87	0.043	3.10	2.27–4.22	0.000
Child in need status (yes) ^e^	1.30	0.85–1.98	0.220	0.75	0.51–1.12	0.160	1.93	1.27–2.92	0.002
**Group**	**4: Medium-decreasing trajectory group**			**4: High-decreasing trajectory group**			**4: Medium-increasing trajectory group**		
Female ^a^	0.78	0.66–0.93	0.005	3.27	2.45–4.37	0.000	0.66	0.49–0.89	0.000
Non-binary or Questioning ^a^	2.75	0.67–11.34	0.161	1.91	0.15–24.51	0.618	2.63	1.01–6.88	0.007
Ethnicity - Asian ^b^	0.43	0.34–0.55	0.000	1.02	0.74–1.40	0.909	0.65	0.38–1.11	0.673
Ethnicity - Black ^b^	0.54	0.40–0.73	0.000	0.37	0.25–0.55	0.000	1.17	0.57–2.38	0.974
Ethnicity - Mixed ^b^	0.65	0.45–0.94	0.022	0.52	0.29–0.91	0.023	1.59	0.92–2.77	0.115
Ethnicity - Other ^b^	0.80	0.55–1.16	0.236	1.13	0.67–1.88	0.648	0.93	0.35–2.48	0.098
Free school meal eligibility (yes) ^c^	2.32	1.98–2.72	0.000	1.59	1.27–1.98	0.000	3.44	2.53–4.68	0.877
Special education need (yes) ^d^	4.17	3.24–5.38	0.000	3.31	2.45–4.48	0.000	2.75	1.74–4.34	0.000
Child in need status (yes) ^e^	1.53	1.09–2.16	0.014	0.86	0.56–1.34	0.513	1.56	0.82–2.95	0.000
**Group**	**5: High-stable trajectory group**			**5: High trajectory group**			**5: High-slightly-decreasing trajectory group**		
Female ^a^	1.59	1.35–1.89	0.000	18.78	14.59–24.17	0.000	0.30	0.24–0.38	0.000
Non-binary or Questioning ^a^	7.54	1.96–29.06	0.003	31.69	5.12–196.28	0.000	0.22	0.04–1.10	0.066
Ethnicity - Asian ^b^	0.11	0.07–0.18	0.000	0.27	0.18–0.40	0.000	0.32	0.20–0.54	0.000
Ethnicity - Black ^b^	0.18	0.11–0.29	0.000	0.15	0.10–0.23	0.000	1.70	1.06–2.72	0.029
Ethnicity - Mixed ^b^	0.48	0.31–0.73	0.001	0.45	0.28–0.72	0.001	1.08	0.64–1.81	0.771
Ethnicity - Other ^b^	0.35	0.20–0.62	0.000	0.36	0.19–0.68	0.002	0.98	0.49–1.96	0.958
Free school meal eligibility (yes) ^c^	3.57	2.98–4.26	0.000	2.30	1.85–2.85	0.000	4.41	3.49–5.58	0.000
Special education need (yes) ^d^	4.26	3.21–5.66	0.000	2.29	1.64–3.19	0.000	4.08	2.87–5.81	0.000
Child in need status (yes) ^e^	1.84	1.28–2.63	0.001	0.87	0.57–1.33	0.516	2.39	1.49–3.84	0.000

*Note. N* = 14,215. OR = odds ratio. CI = confidence interval. ^a^ reference category is male; ^b^ reference category is White; ^c^ reference category is those without eligibility for free school meals; ^d^ reference category is those without special education need status; ^e^ reference category is those without child in need status

### RQ 2: are young women and girls more likely to be represented in trajectory groups with higher levels of, and/or steeper increases in, mental health difficulties?

**Fig. 2:**
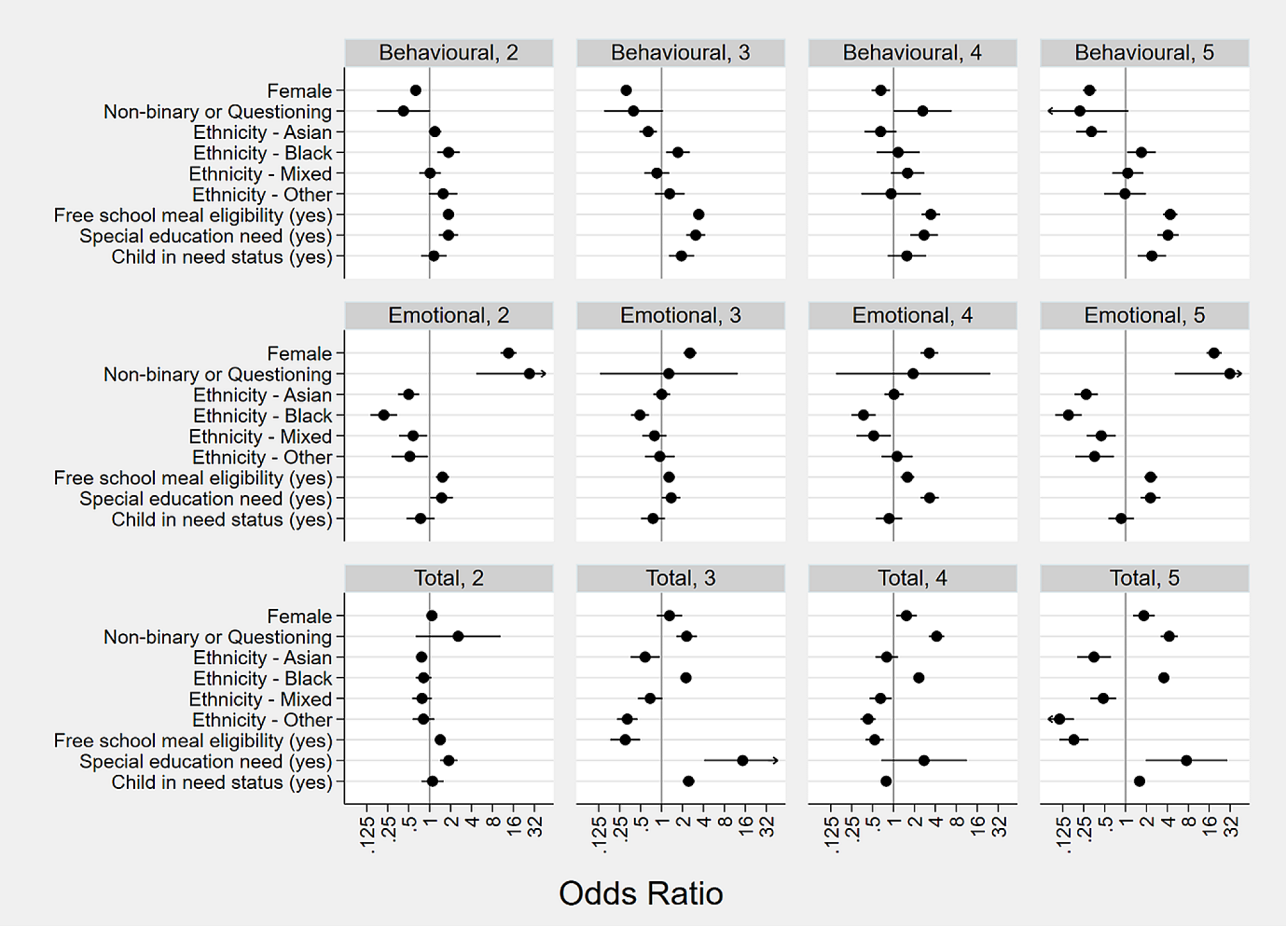
Odds ratios for predictors of mental health trajectory models *Note*. Error bars are 95% Confidence Intervals

In the next stage of the analysis, we added socio-demographic characteristics to the trajectory models as predictors of group membership (see Fig. 2; Table 2).

In terms of total difficulties, compared to trajectory groups with low or medium levels of difficulties, females were more likely to be in the two groups experiencing high levels of difficulties, compared to males. Compared to the low-stable trajectory group, females had 2.45 (95% Confidence Interval or CI: 2.01 to 3.00) times the odds of being in the medium-increasing trajectory group, compared to males. Compared to the low-stable trajectory group, females were 60% more likely to be in the high-stable trajectory group, and 20% less likely to be in the high-decreasing trajectory group, compared to males.

Compared to low or medium levels of difficulties in the third year, non-binary or questioning children and young people were also more likely to be in the two groups experiencing high levels of difficulties, compared to males. It should be noted that the confidence intervals for non-binary and questioning children and young people are generally large due to the relatively small sample size.

For emotional difficulties, compared to the low-stable trajectory group, females were more likely to be in any of the trajectory groups experiencing difficulties, compared to males. There were particularly large effects for two of the trajectory groups: compared to the low-stable trajectory group, females had 13.7 (95% CI 10.5–17.8) times the odds of being in the medium-increasing trajectory group, and 18.8 (95% CI 14.6–24.2) times the odds of being in the high-stable trajectory group, compared to males. Compared to the low-stable trajectory group, non-binary or questioning children and young people were more likely to be in the medium-increasing or high-stable trajectory groups, compared to males.

In terms of behavioural difficulties, compared to the low-stable trajectory group, females were less likely be in any of the trajectory groups experiencing difficulties, compared to males. Compared to the low-stable trajectory group, non-binary or questioning children and young people had 2.6 (95% CI 1.0–6.9) times the odds of being in the medium-increasing trajectory group, compared to males.

### RQ 3: which marginalised identities are more likely to be represented in trajectory groups with higher levels of mental health difficulties?

In instances where ethnicity showed significant effects, the trends indicated that, in comparison to trajectory groups characterised by lower total and emotional difficulties, children and young individuals from black and minoritised ethnic backgrounds were less likely to belong to trajectory groups with elevated difficulty levels, as opposed to their white ethnic counterparts.

Compared to the low-stable total and behavioural difficulties trajectory groups, children and young people with free school meal eligibility, special educational needs, or child in need status were consistently more likely to be in trajectory groups with higher levels of total or behavioural difficulties, compared to children without these additional needs.

Compared to the low-stable emotional difficulties trajectory group, children and young people with free school meal eligibility or special educational needs were consistently more likely to be in trajectory groups with higher levels of emotional difficulties, compared to children and young people without these additional needs.

### RQ 4: is there evidence for intersectional effects between gender and membership of other marginalised groups?

The results showed that the interaction terms between gender identity and the other socio-demographic characteristics were generally not significant (Table S2, available online). This implies that the effects of mental health disadvantage for these multiply marginalised identities in the present sample were additive rather than multiplicative from a statistical perspective.

To facilitate interpretation of the effects for multiply marginalised identities, estimated probabilities of total difficulties trajectory group membership from the trajectory models are presented in Figure S3 (available online). These illustrate that females and males without free school meal eligibility, special education needs, and child in need status had a 28–30% likelihood of being in the low-stable total difficulties trajectory group, whereas females and males from any of these marginalised groups had a 6–7% likelihood of being in the low-stable total difficulties trajectory group.

## Discussion

The aim of the present study was to answer the overarching research question: What are the mental health trajectories for young women and girls? We conducted a secondary analysis of a recent, large-scale community mental health dataset collected over three years [34]. Except for behavioural difficulties, females and non-binary and questioning children and young people belonged to trajectory groups with higher levels of mental health difficulties in England.

These findings are consistent with previous research showing: (a) higher levels of mental health difficulties for young women and girls, (b) higher levels of emotional difficulties for young women and girls, and (c) higher levels of behavioural difficulties for young men and boys [2, 4, 42]. Such differences are unsurprising given the heteronormative gender socialisation of the ways in which distress is expressed. Moreover, considering that girls and young women enter pubertal status earlier may create additional risk as it reflects greater social and emotional challenge for a younger person less able to handle such changes [43]. In line with Borsboom’s network theory of psychopathology [32], this study has shown that difficulties accumulated increase the risk of adolescents’ likelihood of having higher mental health difficulties; moreover, as risks trigger each other, the severity of mental health difficulties also increase.

Despite the availability of national statistics on adult gender diversity and sexual orientation in England, comparable figures for young people are currently lacking. However, the rates of individuals identified as non-binary and questioning in this study was comparable to the most recent census among those aged 16 years and over [44]. Compared to another UK based study among adolescents [45], the non-binary and questioning group in the present data was slightly smaller but this might have been due to data collection methodology/location. Considering the small number of individuals identified as non-binary and questioning, the precision of the estimates for this group is limited. Non-binary and questioning children and young people were also more likely to be in groups experiencing medium (and increasing) and high level of emotional and total difficulties, and medium (and increasing) levels of behavioural difficulties, compared to young men and boys. These findings are consistent with research showing that non-binary individuals experience higher levels of mental health difficulties compared to cis and trans men and women [e.g., 46, 47]. A recent descriptive analysis of the HeadStart sample highlighted that cisgender young people were most likely to have higher subjective wellbeing and lower mental health difficulties and report having high levels of support. On the other hand, non-binary young people, transgender young people and young people who were questioning their identity had lower subjective wellbeing and higher levels of mental health difficulties [48]. According to minority stress theory, health disparities result from exposure to unique forms of stress and adversity arising from oppression, structural inequity, and systemic discrimination [28, 29]. Over time, these experiences may interact with internal thoughts and feelings, resulting in the anticipation or expectation of discrimination or rejection [49]. In turn, this may lead to hypervigilance toward threat and pressure to conceal one’s identity to protect from harm.

The findings of the present research showed that children and young people with free school meal eligibility, special education needs, and/or a child in need status were generally more likely to experience higher levels of mental health difficulties. This effect was seen for young women and girls and young men and boys. This meant that for young women and girls, who were already experiencing higher levels of mental health difficulties, distress was further exacerbated if they were also in groups with any of these additional needs. These findings are in line with an intersectional perspective in that individuals from multiple marginalised groups are exposed to distinct additional disadvantages for each marginalised group to which they belong [30]. White young people were more likely to experience higher levels of mental health difficulties than those from black and Asian backgrounds. This is in line with the previous longitudinal findings [50, 51]. Further research is needed to examine whether this is a difference in prevalence, a difference in questionnaire interpretations and responses, and/or an indication that more mental health measures developed with young people from minoritised ethnic groups is needed.

It is important to note the methodological limitations of the study. First, the sample was not drawn to be representative of all children and young people in England. However, the participants were from six local areas of England spanning different geographic regions, which may increase the generalisability of the results. Second, gender identity was mainly extracted from the NPD, which only captures male and female identities, and only a small proportion of children and young people self-reported gender identity. It is important that future studies include self-reported gender identity to overcome the restricted view from administrative data. Third, even though self-report is an acknowledged way of measuring mental health, it can be subject to limitations such as social desirability [52]. Fourthly, some of the interaction terms (such as for non-binary or questioning young people with special education needs) were very small as indicated by the very large confidence intervals. More confirmatory work with larger samples would be useful. Finally, the findings of the present research do not explain why we observed different patterns of mental health. Qualitative work could further investigate gender differences in mental health, helping us to understand the mechanisms underpinning the findings of the present study.

The findings of the present research show that young women and girls experience higher levels of emotional and total difficulties than young men and boys. There was a similar pattern for non-binary and questioning children and young people. Children and young people with additional needs (eligible for free school meals, special educational needs, or a child in need status) also experienced higher levels of mental health difficulties, an effect that further exacerbated levels of distress experienced by young women and girls. Public mental health approaches that are personalised to individual needs are urgently needed with an immediate priority being to support young women and girls, non-binary and questioning children and young people, and those from other marginalised groups such as individuals from ethnic minority groups.

Future studies should include self-reported gender identity to broaden perspectives beyond administrative data and to better understand and address inequalities.

### Electronic supplementary material

Below is the link to the electronic supplementary material.

Supplementary Material 1

## Data Availability

HeadStart data cannot be made publicly available, since consent was not obtained from participants for the public sharing of their survey responses. However, an anonymised version of the survey dataset used in the present paper is available on request from the corresponding author under the following terms: (1) Schedule and arrange for site visit to AF to analyse data (password to user account supplied). (2) Analysis to be worked on in situ. (3) Results (but not data) taken away.

## Abbreviations

BIC: Bayesian Information Criterion
NPD: National Pupil Database
LGBTQIA+: Lesbian, gay, bisexual, transgender, questioning, intersex, asexual, and other
SDQ: Strengths and Difficulties Questionnaire
