# Supplementary material for: Gender, marginalised groups, and young people’s mental health: a longitudinal analysis of trajectories
Source: Child Adolesc Psychiatry Ment Health. 2024 Feb 28;18:29. doi: 10.1186/s13034-024-00720-4 (PMC10902968; doi:10.1186/s13034-024-00720-4)
Supplement: Supplementary file 1 — Supplementary Material 1 [file 13034_2024_720_MOESM1_ESM.docx]

| **Supplementary Table 1: Fit indices** | | | | | | | |
| --- | --- | --- | --- | --- | --- | --- | --- |
| **Outcome Variables** | **Class** | **BIC** | **Entropy** | **Outcome Variables** | **Class** | **BIC** | **Entropy** |
| Total  Difficulties | 1 | 126921.84 |  |  |  |  |  |
|  | 2 | 121973.17 | 0.719 |  |  |  |  |
|  | 3 | 120644.49 | 0.700 |  |  |  |  |
|  | 4 | 120389.90 | 0.650 |  |  |  |  |
|  | 5 | 120171.25 | 0.640 |  |  |  |  |
|  | 6 | 120088.62 | 0.610 |  |  |  |  |
|  | 7 | 119966.16 | 0.643 |  |  |  |  |
|  | 8 | 119888.39 | 0.660 |  |  |  |  |
|  | 9 | 119889.53 | 0.617 |  |  |  |  |
| Emotional  Difficulties | 1 | 90636.08 |  | Behavioural  Difficulties | 1 | 80285.44 |  |
|  | 2 | 86626.58 | 0.683 |  | 2 | 76808.88 | 0.653 |
|  | 3 | 85804.23 | 0.634 |  | 3 | 75747.33 | 0.687 |
|  | 4 | 85629.18 | 0.628 |  | 4 | 75565.86 | 0.668 |
|  | 5 | 85529.73 | 0.583 |  | 5 | 75484.69 | 0.680 |
|  | 6 | 85477.78 | 0.604 |  | 6 | 75426.41 | 0.667 |
|  | 7 | 85423.52 | 0.705 |  | 7 | 75373.82 | 0.658 |
|  | 8 | 85415.85 | 0.756 |  | 8 | 75404.87 | 0.700 |
|  | 9 | 85359.69 | 0.756 |  | 9 | 75286.71 | 0.655 |

| **Supplementary Table 2: Total difficulties with interaction terms** | | | | | |  |  |
| --- | --- | --- | --- | --- | --- | --- | --- |
| Predictors | OR | 95% CI | | P-value | | |  |
| Group 2 (Medium trajectory group) | |  |  | |  | | |
| Female ^a^ | 1.07 | 0.95 – 1.21 | | 0.257 | | |  |
| Ethnicity - Black ^b^ | **0.64** | **0.49 – 0.83** | | **0.001** | | |  |
| Ethnicity - Asian ^b^ | **0.69** | **0.55 – 0.90** | | **0.001** | | |  |
| Ethnicity - Mixed ^b^ | **0.65** | **0.47 – 0.99** | | **0.011** | | |  |
| Ethnicity - Other ^b^ | **0.69** | **0.48 – 1.66** | | **0.042** | | |  |
| Free school meal eligibility (yes) ^c^ | **1.43** | **1.23 – 1.66** | | **0.000** | | |  |
| Special education need (yes) ^d^ | **1.97** | **1.56 – 2.49** | | **0.000** | | |  |
| Child in need status (yes) ^e^ | 1.20 | 0.83 – 1.73 | | 0.342 | | |  |
| Female x Black | 1.34 | 0.93 – 1.94 | | 0.116 | | |  |
| Female x Asian | 1.18 | 0.89 – 1.56 | | 0.250 | | |  |
| Female x Mixed | 1.43 | 0.90 – 2.26 | | 0.132 | | |  |
| Female x Other | 1.43 | 0.85 – 2.41 | | 0.173 | | |  |
| Female x Free school meal eligibility (yes) | 0.93 | 0.75 – 1.14 | | 0.487 | | |  |
| Female x Special education need (yes) | **0.69** | **0.48 – 1.00** | | **0.048** | | |  |
| Female x Child in need status (yes) | 0.92 | 0.55 – 1.52 | | 0.735 | | |  |
| Group 3 (Medium increasing trajectory group) | |  |  | |  | | |
| Female ^a^ | **1.56** | **1.27 – 1.92** | | **0.000** | | |  |
| Ethnicity - Black ^b^ | **0.33** | **0.18 – 0.62** | | **0.001** | | |  |
| Ethnicity - Asian ^b^ | **0.47** | **0.29 – 0.76** | | **0.002** | | |  |
| Ethnicity - Mixed ^b^ | 0.95 | 0.55 – 1.64 | | 0.843 | | |  |
| Ethnicity - Other ^b^ | **0.24** | **0.08 – 0.73** | | **0.012** | | |  |
| Free school meal eligibility (yes) ^c^ | **1.73** | **1.32 – 2.26** | | **0.000** | | |  |
| Special education need (yes) ^d^ | **2.60** | **1.82 – 3.72** | | **0.000** | | |  |
| Child in need status (yes) ^e^ | 1.14 | 0.62 – 2.09 | | 0.672 | | |  |
| Female x Black | 0.84 | 0.37 – 1.91 | | 0.681 | | |  |
| Female x Asian | 0.71 | 0.39 – 1.29 | | 0.259 | | |  |
| Female x Mixed | 0.65 | 0.30 – 1.40 | | 0.271 | | |  |
| Female x Other | **4.66** | **1.39 – 15.64** | | **0.013** | | |  |
| Female x Free school meal eligibility (yes) | 1.33 | 0.94 – 1.87 | | 0.102 | | |  |
| Female x Special education need (yes) | 0.62 | 0.37 – 1.06 | | 0.079 | | |  |
| Female x Child in need status (yes) | 1.19 | 0.55 – 2.58 | | 0.652 | | |  |
| Group 4 (Medium decreasing trajectory group) | |  |  | |  | | |
| Female ^a^ | 0.95 | 0.83 – 1.10 | | 0.508 | | |  |
| Ethnicity - Black ^b^ | **0.36** | **0.26 – 0.50** | | **0.000** | | |  |
| Ethnicity - Asian ^b^ | **0.41** | **0.31 – 0.54** | | **0.000** | | |  |
| Ethnicity - Mixed ^b^ | **0.56** | **0.38 – 0.83** | | **0.003** | | |  |
| Ethnicity - Other ^b^ | 0.74 | 0.50 – 1.11 | | 0.149 | | |  |
| Free school meal eligibility (yes) ^c^ | **2.14** | **1.81 – 2.53** | | **0.000** | | |  |
| Special education need (yes) ^d^ | **3.29** | **2.57 – 4.20** | | **0.000** | | |  |
| Child in need status (yes) ^e^ | **1.62** | **1.10 – 2.39** | | **0.014** | | |  |
| Notes: ^a^ reference category is male; ^b^ reference category is White; ^c^ reference category is those without eligibility to free school meals; ^d^ reference category is those without special education need status; ^e^ reference category is those without child in need status. | | | | | | |  |
| **Supplementary Table 2 Cont.** |  |  | |  | | |  |
| Predictors | OR | 95% CI | | P-value | | |  |
| Female x Black | **1.79** | **1.14 – 2.81** | | **0.012** | | |  |
| Female x Asian | 1.09 | 0.75 – 1.58 | | 0.647 | | |  |
| Female x Mixed | 1.43 | 0.84 – 2.44 | | 0.186 | | |  |
| Female x Other | 1.08 | 0.59 – 1.95 | | 0.805 | | |  |
| Female x Free school meal eligibility (yes) | 1.00 | 0.79 – 1.26 | | 0.973 | | |  |
| Female x Special education need (yes) | 0.84 | 0.57 – 1.23 | | 0.367 | | |  |
| Female x Child in need status (yes) | 0.81 | 0.47 – 1.40 | | 0.454 | | |  |
| Group 5 (High trajectory group) | |  |  | |  | | |
| Female ^a^ | **1.26** | **1.04 – 1.54** | | **0.021** | | |  |
| Ethnicity - Black ^b^ | **0.23** | **0.13 – 0.41** | | **0.000** | | |  |
| Ethnicity - Asian ^b^ | **0.19** | **0.11 – 0.33** | | **0.000** | | |  |
| Ethnicity - Mixed ^b^ | **0.58** | **0.34 – 0.98** | | **0.044** | | |  |
| Ethnicity - Other ^b^ | **0.44** | **0.23 – 0.83** | | **0.011** | | |  |
| Free school meal eligibility (yes) ^c^ | **2.27** | **1.81 – 2.86** | | **0.000** | | |  |
| Special education need (yes) ^d^ | **4.49** | **3.37 – 5.99** | | **0.000** | | |  |
| Child in need status (yes) ^e^ | **2.07** | **1.31 – 3.27** | | **0.002** | | |  |
| Female x Black | 0.84 | 0.38 – 1.84 | | 0.659 | | |  |
| Female x Asian | 0.70 | 0.35 – 1.43 | | 0.327 | | |  |
| Female x Mixed | 0.85 | 0.40 – 1.78 | | 0.660 | | |  |
| Female x Other | 0.81 | 0.32 – 2.01 | | 0.647 | | |  |
| Female x Free school meal eligibility (yes) | **1.59** | **1.17 – 2.16** | | **0.003** | | |  |
| Female x Special education need (yes) | **0.53** | **0.34 – 0.83** | | **0.006** | | |  |
| Female x Child in need status (yes) | 0.80 | 0.43 – 1.48 | | 0.475 | | |  |
| Notes: ^a^ reference category is male; ^b^ reference category is White; ^c^ reference category is those without eligibility to free school meals; ^d^ reference category is those without special education need status; ^e^ reference category is those without child in need status. | | | | | | |  |

|  |  |
| --- | --- |
|  |  |
| Supplementary Figure 1: Mental Health Difficulties 3-class models | |

Notes: Year 7 (aged 11/12); Year 8 (aged 12/13); Year 9 (aged 13/14)

|  |  |
| --- | --- |
| Supplementary Figure 2: 5-class models for girls and boys | |

Supplementary Figure 3: Comparison between those with adversities vs. not
